# Supplementary figures and images for: Structure of the human CTF18−RFC clamp loader bound to PCNA
Source: eLife. 2026 Feb 23;13:RP103493. doi: 10.7554/eLife.103493 (PMC12928700; doi:10.7554/eLife.103493)

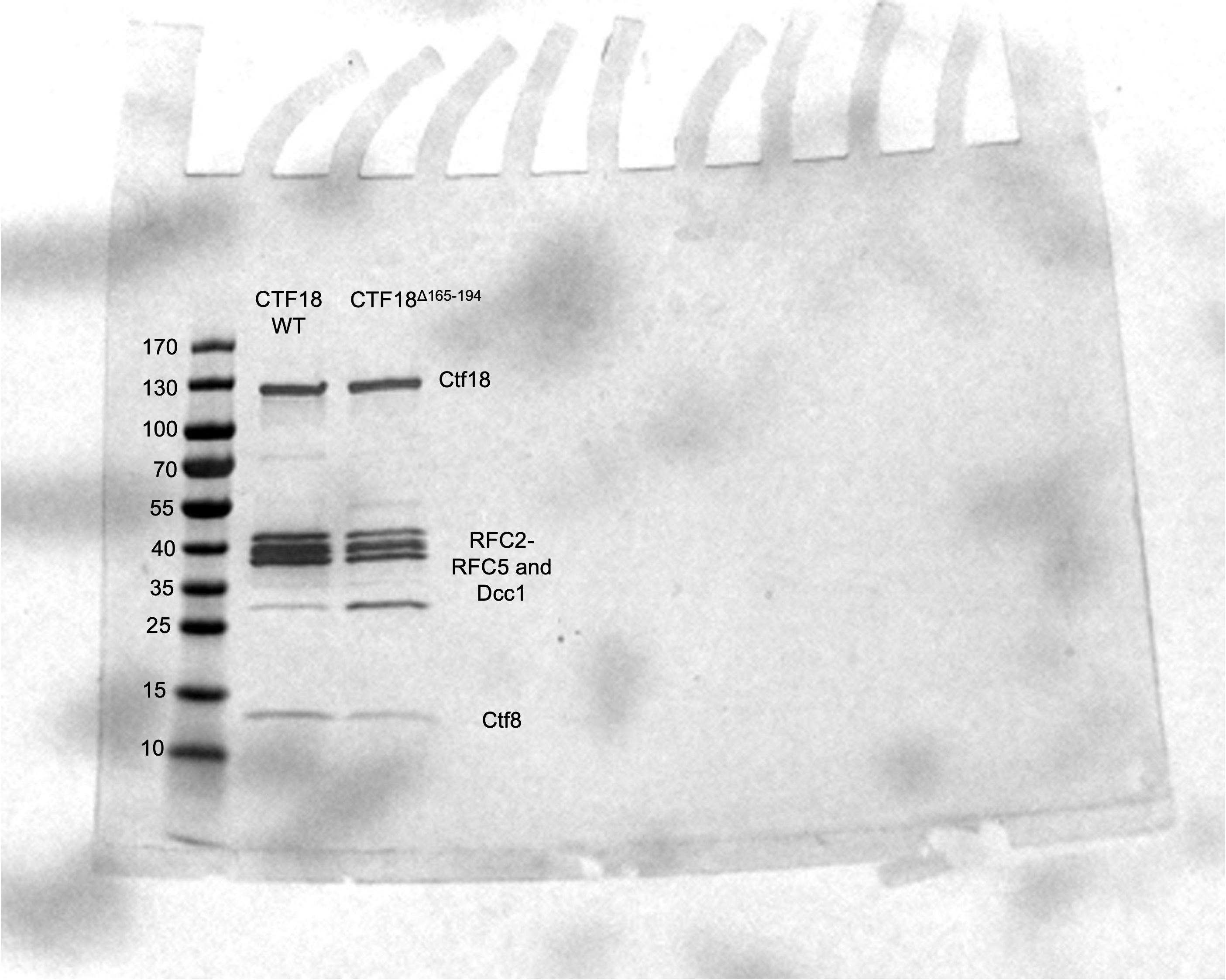

Supplement: Figure 1—figure supplement 1—source data 1. [file elife-103493-fig1-figsupp1-data1.tiff]

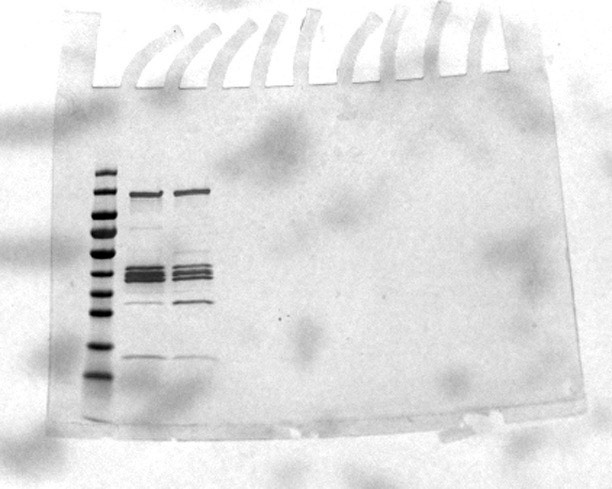

Supplement: Figure 1—figure supplement 1—source data 2. [file elife-103493-fig1-figsupp1-data2.tiff]

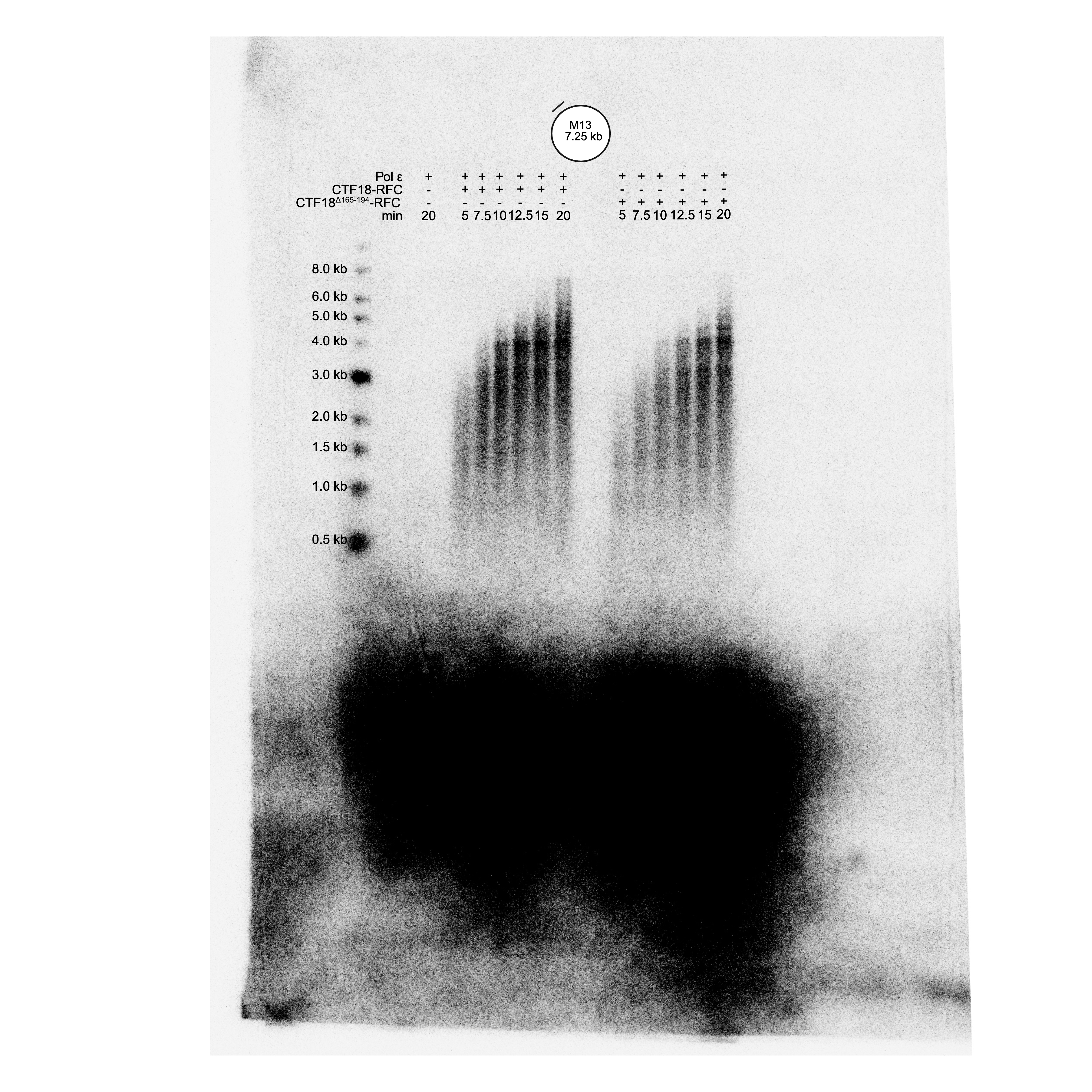

Supplement: Figure 7—source data 1. [file elife-103493-fig7-data1.zip › Figure7_source_Data_raw_uncropped_labeled/Figure7_Source_Data_1.tiff]

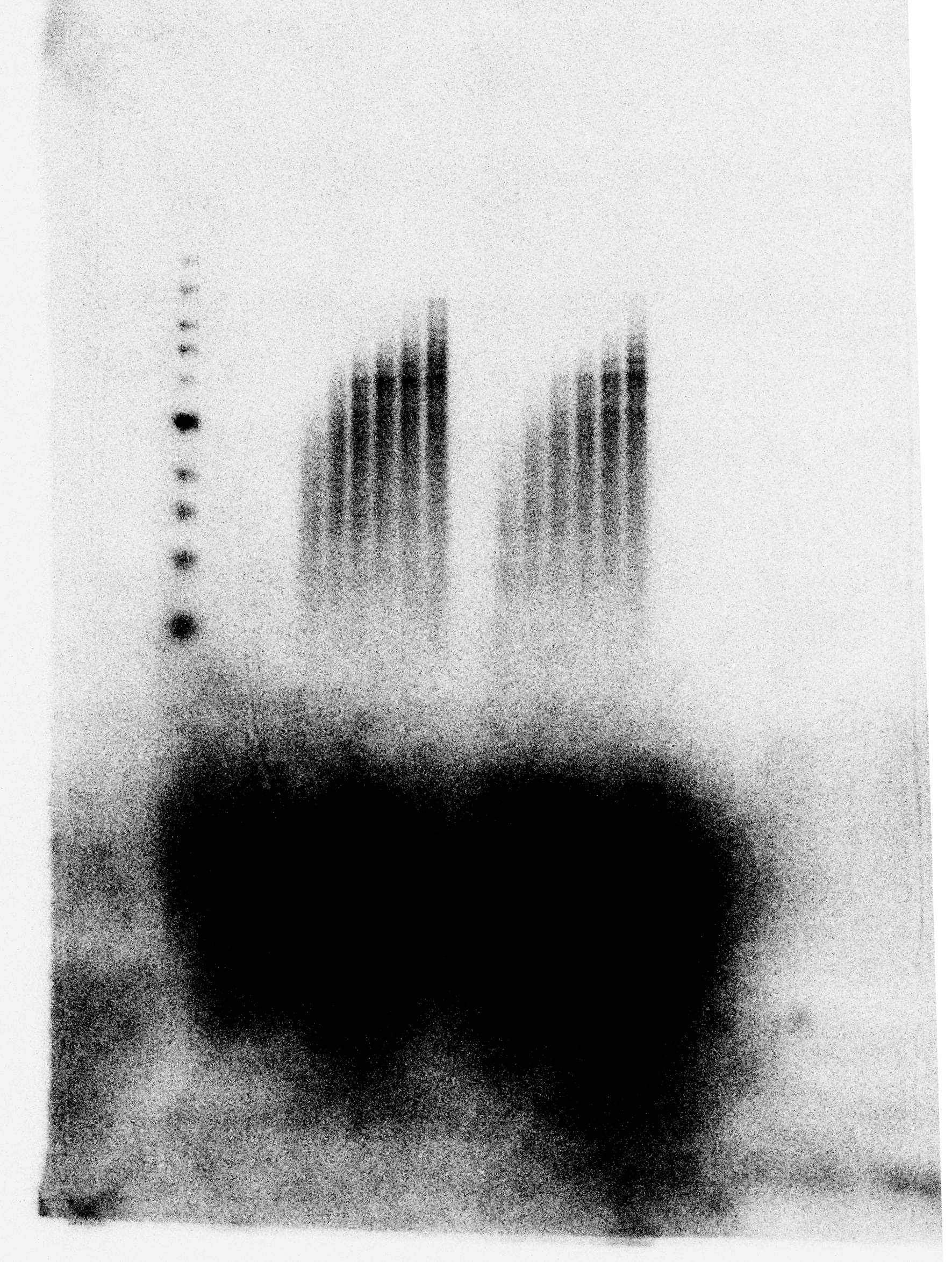

Supplement: Figure 7—source data 2. [file elife-103493-fig7-data2.zip › Figure7_source_Data_raw_uncropped_unlabelled/Figure7_Source_Data_2.tiff]

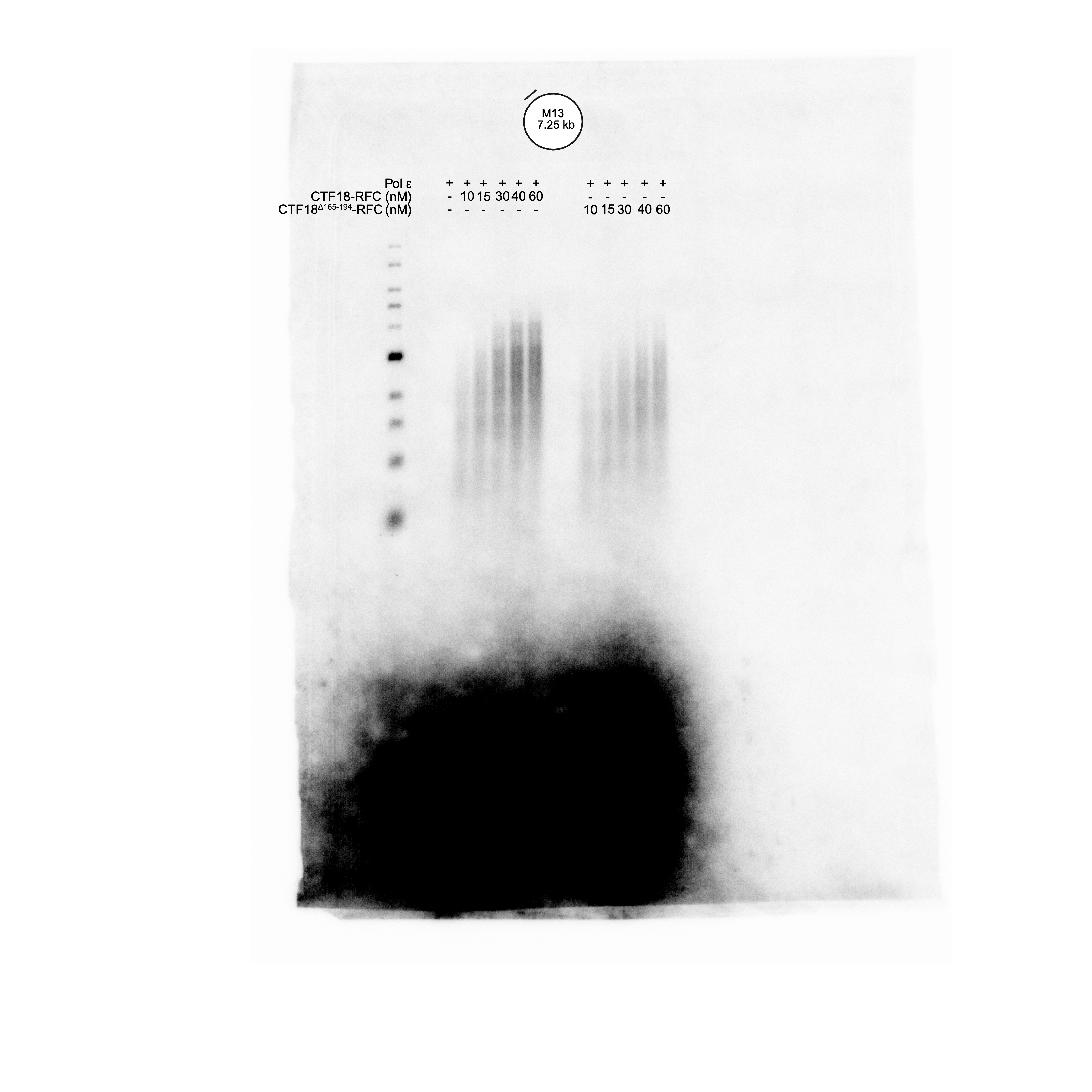

Supplement: Figure 7—figure supplement 1—source data 1. [file elife-103493-fig7-figsupp1-data1.zip › Figure7_Supplement_1_raw_uncropped_labelled/Figure7_Supplement1_Source_Data_1.tiff]

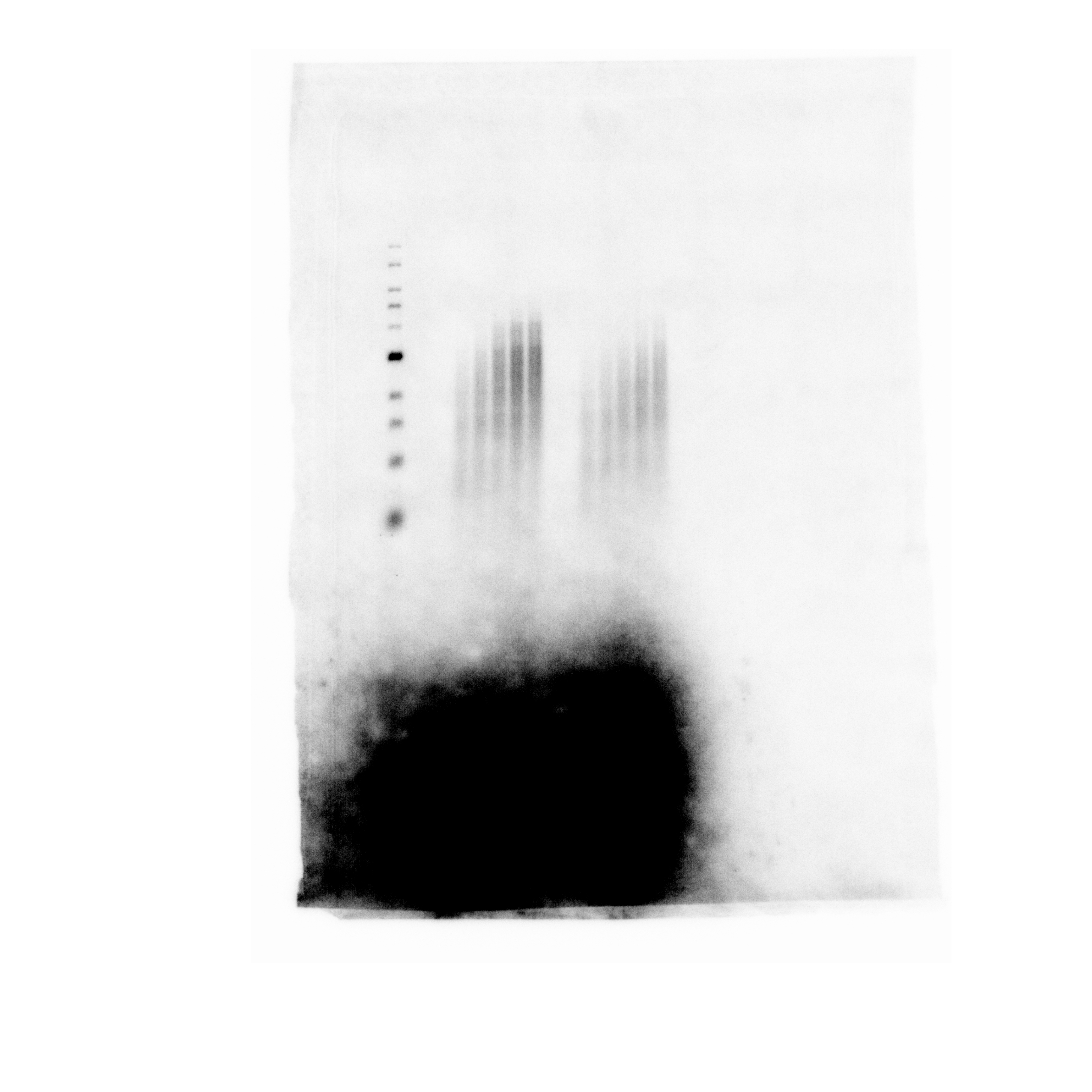

Supplement: Figure 7—figure supplement 1—source data 2. [file elife-103493-fig7-figsupp1-data2.zip › Figure7_Supplement_1_raw_uncropped_unlabelled/Figure7_Supplement1_Source_Data_2.tiff]
