# Supplementary material for: Structure of the human CTF18−RFC clamp loader bound to PCNA
Source: eLife. 2026 Feb 23;13:RP103493. doi: 10.7554/eLife.103493 (PMC12928700; doi:10.7554/eLife.103493)
Supplement: Supplementary file 1. [file elife-103493-supp1.docx]

|  | CTF18-PCNA  with ATP | CTF18-PCNA  with ATP and Mg^2+^ |
| --- | --- | --- |
| **Data collection and processing** |  |  |
| Magnification | 130,000 | 130,000 |
| Voltage (kV) | 300 | 300 |
| Electron exposure (e–/Å^2^) | 43 | 46 |
| Defocus range (μm) | -2.7 to -1.5 | -2.7 to -1.5 |
| Pixel size (Å) | 0.93 | 0.93 |
| Symmetry imposed | C1 | C1 |
| Initial particle images (no.) | 2,043,120 | 3,216,167 |
| Final particle images (no.) | 527,527 | 602,591 |
| Map resolution (Å)  FSC threshold | 2.9  0.143 | 3.3  0.143 |
| Map resolution range (Å) | 2.8-3.8 | 3.1-4.3 |
|  |  |  |
| **Refinement** |  |  |
| Initial model used (PDB code) | 6VVO | 6VVO |
| Model resolution (Å)  FSC threshold | 3.0  0.5 | 3.5  0.5 |
| Map sharpening *B* factor (Å^2^) | -67.879 | -138.637 |
| Model composition  Non-hydrogen atoms  Protein residues  Ligands | 19777  2503  4 | 19779  2503  6 |
| *B* factors (Å^2^)  Protein  Ligand | 68.48  80.11 | 169.79  167.64 |
| R.m.s. deviations  Bond lengths (Å)  Bond angles (°) | 0.004  0.612 | 0.003  0.547 |
| Validation  MolProbity score  Clashscore  Poor rotamers (%) | 2.26  11.75  3.47 | 2.00  9.39  2.19 |
| Ramachandran plot  Favored (%)  Allowed (%)  Disallowed (%) | 96.14  3.82  0.04 | 96.38  3.49  0.12 |
